# Can the diagnostic accuracy of newborn eye screening for congenital cataract be improved with digital imaging? The Digital Imaging versus Ophthalmoscopy (DIvO) study

**DOI:** 10.3310/nihropenres.13370.1

**Published:** 2023-05-12

**Authors:** Louise Allen, Catherine Bain, Lindsey Rose, Simon Bond, Jugnoo Rahi

**Affiliations:** 1 Ophthalmology Department, Cambridge University Hospitals NHS Foundation Trust, Cambridge, N/A, CB2 0QQ, UK; 2 Cambridge Clinical Trials Unit, Cambridge University Hospitals NHS Foundation Trust, Cambridge, CB2 0QQ, UK; 3 Midwifery Department, Anglia Ruskin University, Cambridge, CB1 1PT, UK; 4 Department of Ophthalmology, Great Ormond Street Hospital for Children, London, UK; 5 Population , Policy and Practice Dept, UCL GOS Institute of Child health, London, WC1N 1EH, UK

**Keywords:** congenital cataract, NIPE, screening, infrared imaging, red reflex, newborn eye examination, digital imaging

## Abstract

**Background:**

Congenital cataract is the commonest cause of preventable child blindness in the world. It affects 1: 3000 babies, 60% of whom will have bilateral disease. Although screening of the newborn red-reflex with an ophthalmoscope is recommended, there are questions over this technique's accuracy, particularly in non-specialist hands. Several studies in enriched paediatric eye clinic populations have demonstrated superior accuracy when a digital image of the the eye's reflection to infrared light is evaluated.

**Aim:**

This study uses a prototype infrared digital camera to test the hypothesis that the sensitivity and specificity of newborn screening using evaluation of the "infrared-reflex" image is superior to the standard red-reflex examination using ophthalmoscopy.

**Methods:**

140,000 newborn babies will be recruited into the study from at least 13 maternity units in England over an 18 month period. Babies will have both the standard red-reflex assessment and evaluation of the infrared-reflex using a prototype device. Since specialist gold-standard evaluation of every participant is impractical, bespoke data linkage requests to NHS England will be made. Data from the red-reflex evaluation and the presence of codes relating to cataract diagnosis and/or treatment in Hospital Episode Statistics (HES) will be retrieved for each participant a minimum of 6 months after their birth. This data will be used to calculate relative and absolute sensitivity and specificity for each screening test and comparison of accuracy using the McNemar test. Secondary outcome measures will include comparison of accuracy in different ethnic groups and screener usability scores.

**Anticipated impact:**

Confirmation of the hypothesis will support development of a commercial screening device and the possible revision of newborn screening recommendations.

**Trial registration:**

The study is registered with clinicaltrials.gov: NCT05282147.

## Plain language summary

Cloudiness of the lens of the eye, called a cataract, is the commonest cause of avoidable child blindness in the world, affecting 1 in 3000 UK infants. Surgery is required within 3 months to prevent permanent visual impairment. Although all babies born in the UK are screened for cataract soon after birth, late diagnosis continues to be a problem.

Eye screening is currently performed by midwives shining a specialist torch (ophthalmoscope) into the eye to assess the red-reflex, like the “red eye” seen in flash photos. Cataract causes a dark shadow on the red-reflex. The test is difficult because bright white light constricts the pupils and makes the baby close their eyes. Assessment of the eye's reflection to infrared light (the "infrared-reflex") has been shown to be more accurate than the red-reflex for detecting cataract in children. Unlike white light, infrared light does not cause pupil constriction and eye closure. A digital camera "neocam" has been specially developed for this study to image the newborn "infrared-reflex".

This study will compare the accuracy of red-reflex and infrared-reflex evaluation in 140,000 newborn babies recruited from at least 13 maternity units across England. All babies will have both the standard screening test and digital eye imaging with the study device. Babies who have a potential abnormality on either screening test will be referred for specialist examination. Hospital records will be used to identify those babies subsequently diagnosed with cataract.

A more accurate screening test could prevent life-long disability and reduce costs to the NHS and society. This study will allow a future estimation of what these savings might be and whether changing to a digital imaging screening service in the UK might be justified. Potentially this could impact eye screening world-wide to prevent childhood blindness from cataract.

## Introduction

### Background and rationale

Congenital cataract is the commonest cause of avoidable childhood blindness worldwide ^1– 3^. Red-reflex screening for congenital cataract is recommended by the UK National Screening Committee within 72 hours of birth and at 6–8 weeks as part of the Newborn and Infant Physical Examination (NIPE). Early surgery for severe cataract is time critical, being required within the first months of life to prevent permanent visual deprivation amblyopia.

Screeners report finding red-reflex assessment difficult because of their unfamiliarity both with rare eye conditions and the technique of ophthalmoscopy ^4^. Additionally, darker ocular pigmentation and the baby’s pupil constriction and aversion reflexes to bright light can limit the quality of the assessment ^5– 8^. A national surveillance study found that only 60% of children having cataract surgery before the age of two years had been referred via the NIPE screening programme ^9^. Conversely, audit data record that referrals to specialist clinics for possible red-reflex abnormalities are 10 times the frequency expected for the prevalence of the condition (Public Health England audit data 2019).

Recent studies have indicated that screener evaluation of the infrared (IR)-reflex image using a prototype camera is superior in accuracy to standard red-reflex assessment in cataract enriched childhood cohorts ^10, 11^. If confirmed in a population screening setting, a more accurate test could minimise the number of babies with cataract whose diagnosis is delayed due to a false negative assessment and reduce the number of unnecessary false positive referrals to specialist clinics and the parental anxiety that causes.

The aim of this study is to confirm or refute the hypothesis that the sensitivity and specificity of screening with IR digital imaging using the neocam prototype device is superior to the standard ophthalmoscopic red-reflex test. This study will provide data for a subsequent health economic analysis to establish if replacing the standard method of screening with IR digital imaging would be cost effective in terms of health outcomes and reduction in unnecessary referrals.

### Review of existing evidence

IR imaging is commonly used in ophthalmology, an advantage being the avoidance of the pupil constriction and aversion response which accompanies flash photography. Additionally, the IR-reflex from the ocular fundus is monochrome, removing the issue of colour variability with ethnic pigmentation which can make evaluation of red-reflex more difficult ^6^. IR photoscreeners are used to detect refractive error and cataract in young children but are not practical for newborn screening because they require visual fixation and a test distance of 1 metre. In 2018, a modified smartphone (Catcam prototype) fitted with co-axial IR illumination was developed for this purpose by the study lead. Two separate proof-of-concept studies (one in Cambridge, the other in Tanzania), confirmed superior accuracy of non-specialist evaluation of the IR-reflex to red-reflex in enriched child clinic populations. With this evidence, the neocam prototype version 1 was developed by a prototyping company 42 Tech Ltd (St Ives, Cambs, UK). This neocam prototype has since been used in over 50,000 children in a large study run by the London School of Hygiene and Tropical Medicine study in Tanzania, Malawi and Uganda.

### The study device

**Figure 1.**
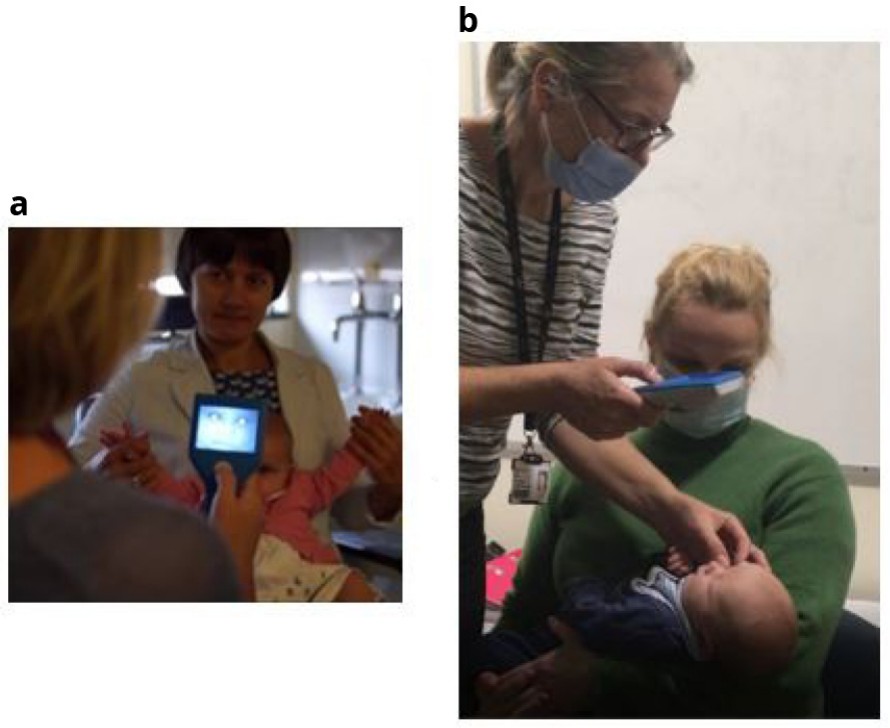
**a**) Neocam in use – the bright infrared-reflexes are clearly visible in this infant **b**) Neocam in use on a newborn baby.

The neocam version 2 prototype is a modified and upcycled version 1 device. It is a hand-held battery powered device, similar in size, weight and appearance to a barcode scanner ( Figure 1a&b). The device is robust and designed to be used in UK normal indoor ambient lighting conditions. Components include an integrated digital camera capturing images illuminated by its co-axial IR LED from a viewing distance of 30cm. An LCD enables data input, real-time view finding and image evaluation. Version 2 includes software and hardware modifications to improve optical resolution and user interface improvements outsourced to Keeler Ltd, (Windsor UK) by the legal manufacturer, Cambridge University Hospitals NHS Trust. The technical file and Investigator Brochure has been submitted to the Medicines and Healthcare Regulatory Authority for approval to be used in this investigation. There is no intention for the current prototype to be commercialised, although a future commercial device will be developed if the study hypothesis is confirmed.

## Objectives and outcome measures

### Primary objective

To estimate and compare the sensitivity and specificity of screening evaluation with the standard test to the intervention digital imaging test in newborn babies.

### Secondary objectives

Comparison of accuracy between tests with respect to ethnicity, comparison of accuracy between tests with respect to screener experience and usability feedback and test preference.

### Primary outcome measure

The primary outcome measures are:

- Screener evaluation “normal” or “abnormality suspected” of each eye using the standard test.
- Screener’s evaluation “normal” or “abnormality suspected” of each eye using the neocam digital imaging test.
- The gold standard evaluation (provided by data linkage +/- expert image review)

### Secondary outcome measure

- Quantitative usability feedback and test preference using a screener questionnaire ^12^.

## Protocol

### Study design

This will be a multi-centre, prospective population-based superiority study. All participants will have both the existing standard test and the intervention test (digital imaging with the neocam device) once only within 72 hours of birth. Evaluations of each test will be compared to the gold-standard.

### Study participants and setting

All newborn babies in maternity units who are eligible for the newborn physical examination. The imaging intervention can be undertaken in the cot or in the mother’s arms.

### Recruitment (see flow chart Figure 2)

**Figure 2.**
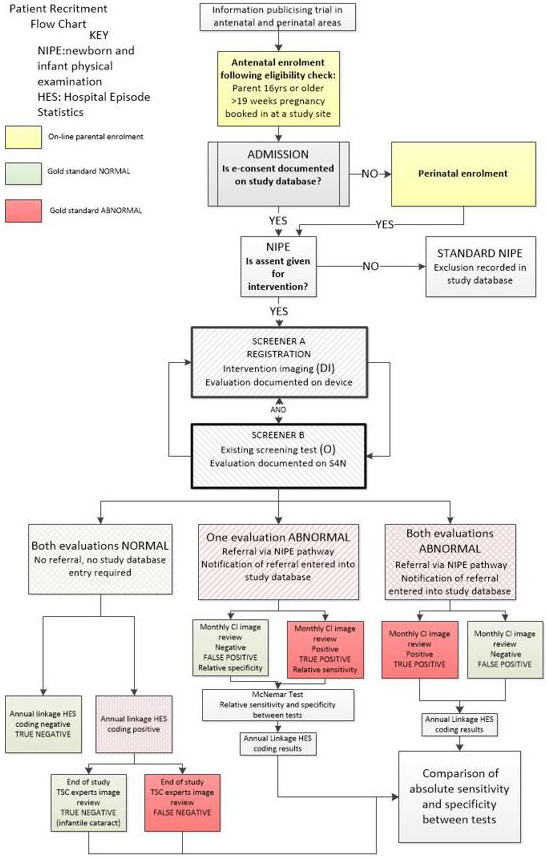
Study flow chart.

Recruitment will take place over at least 18 months in at least 13 large maternity units. Site participation will be prioritised in maternity units with over 4000 births per year and with a diverse ethnic population.

### Enrolment

Parents will be encouraged to enrol from the 20 ^th^ week of pregnancy up until discharge from the maternity unit. Posters and leaflets with a QR code will signpost parents to the study website. Here, the parent will find information about congenital cataract and a short introductory video. The website also hosts patient information leaflets (PIS) in six languages.

The parent is asked to check that they meet several criteria for enrolment:

- They are 16 or over
- They are 19 weeks or more into their pregnancy or within 72 hours post-partum
- They are booked into a participating maternity unit (visible in a drop-down list)

If all criteria are met, a web-button transfers them to the enrolment form in the bespoke secure study database (designed by Sealed Envelope Ltd.).

### e-consent

The negligible risk from study participation enables the use of self-directed informed e-consent. The parent enters their name, NHS number, email address and maternity unit and electronically signs a General Data Protection Regulation (GDPR) statement for this data to be securely retained. A software algorithm checks validity of the maternal NHS number to mitigate against data entry error. After checking that the parent has read and understood the PIS, the parent’s electronic signature is requested on the consent form (eICF). An automated confirmation email with eICF attachment, links to the ePIS and to contact information of the local and central research team are sent to the parent’s email address. Parents will be unable to self-enrol if they do not have an email address. They are advised to seek assistance from their local research team, who can use a hospital device and site NHS email address to complete and print documents with the parent. Following birth, the research team will search the study database for the maternal NHS number to check a copy of the eICF is present. If not, the parent will be invited to enrol and consent at this stage.

### Registration

Once the eICF is documented in the maternal database record, verbal assent is sought prior to digital imaging. The process of the baby’s registration into the study occurs when the clinician inputs the site and user code, maternal NHS number, baby’s NHS number and date of birth (DOB) into the neocam digital imaging device. A software algorithm checks validity of the maternal and baby NHS number to mitigate against data entry error.

### The intervention

After registration, the neocam device is used to image the IR-reflex in each eye. This procedure will be undertaken by a screener who has attended site training, but NIPE and Good Clinical Practice (GCP) certification is not obligatory. The imaging should take place in in either the cot or in the parent’s arms in a normally lit room.

The IR LED illuminates automatically after each eye is selected on the device display. The screener visualises the eye through the device display and aligns a visible ring calliper on the display with the corneal limbus of each eye to promote imaging at an optimal distance. The screener first evaluates the quality of the image (for movement blur and eye closure) and retakes the image if necessary. Imaging will generally take less than 2 minutes in total. Subsequently the screener evaluates each image as “normal” or “abnormality suspected” on the keypad when prompted by the device user interface. This evaluation is stored in the image file name. This ends the baby’s active participation in the study.

### Acquisition of the intervention data

Images from the neocam device are uploaded daily by connecting the device with a Trust computer via a USB cable. The images are copied to the secure study database linked by the maternal NHS number to create the electronic case report form (eCRF). More than one eCRF can be registered under the same maternal record (for instance for multiple births). The images are then deleted from the device itself. In addition to the images, the information uploaded to study database includes: site and user-role code, maternal NHS number, baby’s NHS number, baby’s DOB, date of imaging and the infrared-reflex evaluation for each eye.

### Acquisition of the standard screening data

Red-reflex screening results are routinely inputted into the nationwide Smart4NIPE (S4N) web-based database controlled by NHSE. This data includes the baby’s NHS number, DOB sex, ethnicity, screener role and red-reflex evaluation codes. This data will be retrieved through bespoke data linkage with NHSE records. Any babies with “abnormality suspected” evaluations on their standard screening tests will have this documented in the study database after both screening tests have been completed.

### Acquisition of the gold-standard data (see data linkage Figure 3)

**Figure 3.**
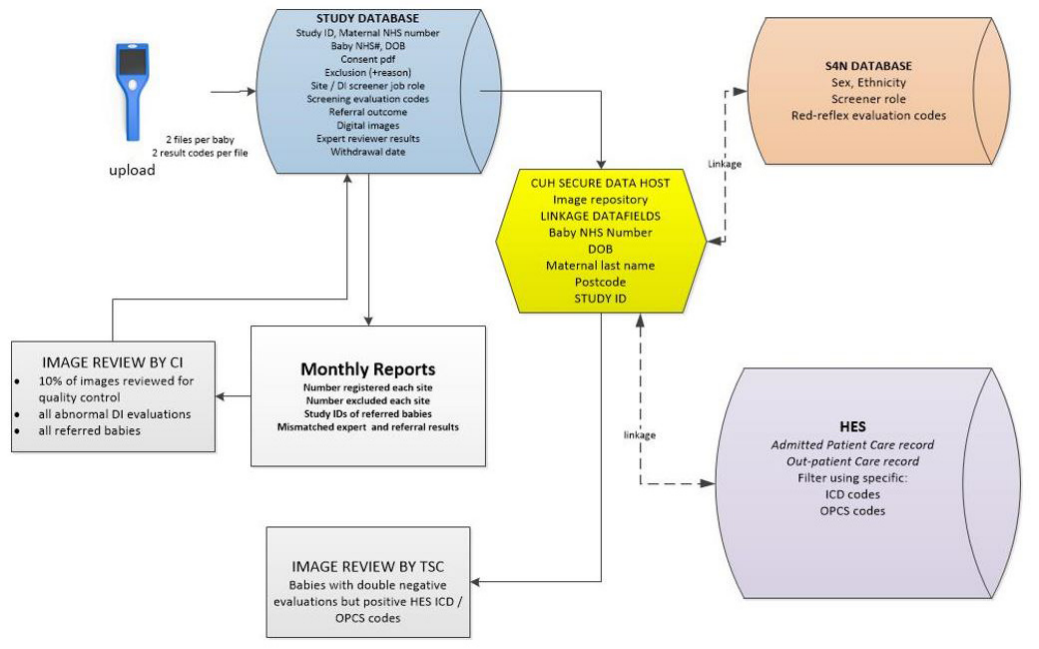
Data linkage chart. Abbreviations: CI: Chief investigator, HES: Hospital Episode Statistics, ICD-10: HES diagnostic codes, OPCS: HES procedure codes, S4N: Smart4NIPE national database, TSC: Trial Steering Committee.

Specialist examination of 140,000 babies for the study to determine “true” disease (cataract) status would be impractical. Therefore, bespoke data linkage with Hospital Episode Statistics (HES) records at least six months after birth will be used to identify registered babies who have subsequently received a specialist diagnosis of cataract (based on both diagnostic and procedure codes). This, with additional expert review of the images when required, will provide the gold standard evaluation of disease status.

- All babies referred for specialist examination (either due to abnormal standard, digital imaging or both) will undergo expert review of the digital images by the Chief Investigator (CI).
- Babies with double negative (evaluated as normal screening tests) but HES evidence of diagnostic/procedure codes indicating cataract will have their digital images reviewed by two independent expert ophthalmologists drawn from the Trial Steering Committee (TSC). This will determine if there was evidence of cataract at birth (which should have been detected by screening) to the gold standard or whether the cataract developed subsequently.
- 10% of all double negative eye images will be routinely reviewed by the CI for quality control purposes. For cases where an abnormality is suspected on image review, a specialty referral will be organised for the baby within two weeks.

### Prevention of result contamination

The standard test and the intervention test will be undertaken by different clinicians. The importance of only sharing the outcome with the parents and other staff once both evaluations have been documented will be stressed in site training. Inadvertent discussions between screeners will result in incident notification in the site file.

### Withdrawal of participants from the study

***Prior to registration.*** This will include babies whose parents do not assent and those without an assigned NHS number. Additionally, if there are insufficient staff to undertake the study intervention or if the device is not working. In all cases, the reason for withdrawal following parent consent will be entered in the study database.

***Following registration.*** Parents may withdraw their consent for data linkage or the use of the images in future development work by contacting the central research team. The reason for withdrawal reason, if given, will be documented on the eCRF in the trial database.

The data and images for registered babies in whom consent has been withdrawn will be retained in the study database and only event data up to the withdrawal date will be collected via data linkage. Given the absence of risk and the single use of the intervention, these numbers are not predicted to be high but further recruitment would be required to replace their loss.

***Usability and screening preference feedback from screeners.*** Screeners undertaking either the standard red-reflex test or neocam imaging will attend site training prior to clinical use. Each will be asked to complete an anonymised usability questionnaire ^12^ during the study period.

***Definition of the end of the trial.*** The end of trial will be 6 months after the last data capture via data linkage.

## Statistics and data analysis plan

### Statistical method

Given the rarity of cataract, and to maximise the data from eyes with cataract captured in the study, the abnormal eye will be selected as the index eye for data analysis where there is a unilateral abnormal screening result. Where the screening test is abnormal bilaterally, the right eye will be selected as the index eye.

A comparison of the relative sensitivity and specificity of the two tests will be possible using the counts of discordant pairs of screening tests, where one, and only one, of the neocam and standard test results within a baby are positive. Thus this relative comparison will be estimable in a short space of time for the recruited population using the expert image review as the gold standard. Hence much of the statistical inference can be performed before the long-term follow-up of the babies with both tests as negative.

For both the groups with and without congenital cataracts (as determined by the gold standard) the sensitivity and specificity, respectively, will be compared. McNemar’s test will provide a p-value, and a 2-sided 2.5% significance level used to account for the two contrasts. The absolute difference and odds ratios will also be estimated and provide with 95% confidence intervals. 

An estimate of the absolute values of, rather than differences between, sensitivity and specificity will be made following TSC expert review of the neocam images of babies with double negative screening tests in whom diagnostic and / or procedure codes indicating cataract were retrieved from HES on data linkage (since the cataract may have developed after birth). A detailed statistical analysis plan will be produced before the final database lock or before any interim analysis is performed.

Equivalent comparisons of sensitivity and specificity for each screening test will be made within ethnic groups and screener experience levels.

Quantitative comparison of the ease of the standard screening examination to the intervention will be analysed by screener questionnaire at the end of that staff member’s rotation on the maternity ward or at the end of the study recruitment period, whichever is sooner ^12^. Summary statistics reporting on the numerical or categorical questions will be provided.

### Number of participants required

From the Cambridge proof-of-concept pilot study using non-specialist screeners, the sensitivity is assumed to be 70% and 95% for red-reflex and digital imaging screening by non-specialist staff respectively. Hence the treatment effect is assumed to be a 25% difference. Power calculations need further assumptions regarding the joint distribution of the test within individual babies; we assume the maximum rate, 35%, of discordant tests consistent with these margins. A 2-sided hypothesis test (McNemar’s) at the 2.5% significance level will have 90% power if 67 babies are recruited with cataracts, necessitating recruitment of 140,000 newborns for screening.

### Criteria for the premature termination of the trial

Poor image quality on reviewed images which is due to technical device issues rather than user error may result in a temporary suspension of the trial in one or all sites to modify the device.

### Procedure to account for missing or spurious data

Participants in whom the S4N or the neocam dataset are missing will be excluded from statistical analysis. Where the quality of scans is insufficient when reviewed by the assigned paediatric ophthalmologist evaluator, the site will receive additional training.

## Data handling and record keeping

All study data will be entered into a secure bespoke web-based data management system designed by Sealed Envelope Ltd. Database entry onto the eCRF within the study database will be undertaken by GCP certified site research or clinical staff. Data will not be editable in the eCRF once the record is saved. The eCRF will be accessible to trial coordinators, data managers, the investigators, Clinical Trial Monitors, Auditors and Inspectors as required.

### Source data

To enable peer review, monitoring, audit and/or inspection, all eCRF data on the research database which will include eCRF, image files and linked data, and completed electronic informed consent forms will be kept securely.

Source data may include but is not limited to:

- Electronic Informed Consent Form
- Relevant sections of the eCRF
- Hospital event data received via data linkage e.g. HES data
- Test result data received via data linkage e.g. S4N data

## Data protection & participant confidentiality

All investigators and study staff will comply with the requirements of the Data Protection Act 1998 and the sponsoring Trust’s Policy with regards to the collection, storage, processing, transfer and disclosure of personal information and will uphold the Act’s core principles.

Participants will provide explicit consent to the use of PID for the purposes of the conduct of the study. PID will be stored and encrypted on a secure NHS server and in compliance with the Data Protection act. Data linkage applications will be made to NHSE to access HES and S4N data. The applications and resulting data will be managed by the Data Transfer Officer (DTO) and coordinating centre at the Cambridge Clinical Trials Unit. Linkage data will be transferred securely in compliance with the Data Protection act. Database access will be restricted to the delegated trial staff.

### Data monitoring committee (IDMEC)/trial steering committee (TSC)

As the inventor of the neocam device, the study lead has potential conflict of interest in the study. To eliminate this, the study leadership will be transferred to the co-lead on completion of the recruitment period. Given the lack of intervention risk and the absence of data analysis during the recruitment period, the NIHR has permitted a subset of independent members on the TSC to additionally fulfil the role of the IDMEC. The TSC comprises experts from the fields of paediatric ophthalmology, neonatology and statistics in addition to the study leads. The group will meet 6 monthly from the initiation of site training to ensure the study is running as planned.

## Ethical and regulatory considerations

### Declaration of Helsinki

The investigator will ensure that the study is conducted in accordance with the principles of the Declaration of Helsinki and in accordance with the relevant regulations and principles of GCP.

### Approvals

The study is registered with clinicaltrials.gov: NCT05282147.

This study has had a favourable opinion from the Research Ethics Committee and Health Research Authority pending neocam device approval for investigation by the Medicines and HealthCare Regulatory Authority. IRAS 293461. If developed, the future device would be classified under the Class 2a category for UKCA / CE marking.

### Expenses and benefits

No additional participant expenses will be expected or remunerated.

### Finance and insurance

This study is funded by an NIHR Efficacy and Mechanism Evaluation grant NIHR 130628 assigned to Louise Allen, Cambridge University Hospitals NHS Trust.

NHS bodies are legally liable for the negligent acts and omissions of their employees. If subjects are harmed whilst taking part in a clinical study resulting from negligence on the part of a member of the study team this liability cover would apply. Cambridge University Hospitals NHS Trust is the legal manufacturer of the neocam prototype device and will hold product liability insurance for its use during this study.

## Study status

Awaiting MHRA approval for investigational use of the neocam device prior to site training.

## Dissemination

Study findings will be disseminated in national and international conferences and open access journals. The findings of the study will be presented to the UK National Screening Committee to inform the committee’s recommendations for newborn eye screening policy.

## Data Availability

No data are associated with this article. Open Science Framework: DIvO study, https://doi.org/10.17605/OSF.IO/NYZW7 ^12^. This project contains the following extended data: DIvo usabilityquestionnairev7.pdf Data are available under the terms of the Creative Commons Zero "No rights reserved" data waiver (CC0 1.0 Public domain dedication).
