# Supplementary material for: Can the diagnostic accuracy of newborn eye screening for congenital cataract be improved with digital imaging? The Digital Imaging versus Ophthalmoscopy (DIvO) study
Source: NIHR Open Res. 2023 May 12;3:25. [Version 1] doi: 10.3310/nihropenres.13370.1 (PMC13264687; doi:10.3310/nihropenres.13370.1)
Supplement: Peer review history

# Reviewer report: Reviewer response for version 1

**DOI:** 10.3310/nihropenres.14501.r29283

**Published:** 2023-06-12

**Authors:** Alistair R. Fielder

**Affiliations:** 1 Department of Optometry & Visual Science, City University of London, London, England, UK

The identification of developmental cataract is unsatisfactory with many not being identified for many months. Worldwide, this consumes vast amounts human and financial resources, thus a simple method which robustly identifies cataract in the neonatal period will be welcomed by all.

 The incidence is low and if my calculations are correct the investigators hope to capture about 50 with cataracts. It is good to see that the results will be the subject of a health economic analysis - in my opinion - critical as this is a high risk study

 Digital imaging presents an opportunity to explore this topic non invasively. I recall a study from China using the RetCam - not a brilliant study but quite large numbers.

**Timing of measurements.** Stated to be within 72 hours of birth. But most babies are discharged to home within 24 hours. I am concerned that this will present several logistical problems: parents and staff will be busy and will naturally be occupied with other thoughts. Also at this time the eyelids are puffy so opening them for the assessment is not as simple as it might seem. A better time would be at the 6-8 week examination but by this time babies are not in hospital. The authors refer to the need for early surgery, but evidence from Eileen Birch and others indicates that the critical period is a little longer than previously thought - thus surgery at 6-8 weeks would not compromise outcome - I am just floating this idea because taking readings within 24 hours could be a problem.

**The two tests. **The baby will be assessed using both (traditional) direct ophthalmoscopy and the neocam device. It is not stated whether both are taken by the same individual or in which order they are taken. It is important that the two readings are taken by different individuals and that there is no communication between them. If each tester is "blind" to the other, perhaps the test order is not important - for consideration

 There is no mention of false positives - these are well known to occur using iPhones (etc). 

 Is it possible that both methods produce false negative results? This is mentioned in the application but it is not mentioned how it would come to light. Follow-up examination is clearly not feasible. Perhaps the results of the routine child surveillance assessments at 8 and 12 weeks and later would provide this information.

**"Bench" testing.** What is the effect of the neocam being off-axis - that is off the line of sight? I cannot gain a sense of this from Duret et al (Eye 2019). It would be interesting - and possibly important to know - what is the effect of the neocam being tilted and off-axis on the identification of lens opacities at their various locations (anterior polar etc). My guess is that this not important, but need to know.

 This is an important study and one which will have implications for clinical practice worldwide. There are a few methodological issues to iron out. I am concerned about the practicalities of obtaining two readings within 24 hours of birth. A health economic analysis is critical.

Is the study design appropriate for the research question?

Yes

Is the rationale for, and objectives of, the study clearly described?

Yes

Are sufficient details of the methods provided to allow replication by others?

Partly

Are the datasets clearly presented in a useable and accessible format?

Yes

Reviewer Expertise:

paediatric ophthalmology, particular neonatal work - retinopathy of prematurity. Also Amblyopia

I confirm that I have read this submission and believe that I have an appropriate level of expertise to confirm that it is of an acceptable scientific standard, however I have significant reservations, as outlined above.

# Response

**Published:** 2023-06-15

**Authors:** Louise Allen

**Affiliations:** Cambridge University Hospitals NHS Foundation Trust, UK

The authors thank Professor Fielder for his review of the study protocol and for the important points he makes.

 For statistical power, we need to recruit 67 babies with cataract and this may require the screening of more than 140,000 babies. Being able to review the neocam images of eyes with a suspected abnormality on screening will enable us to keep a tally of cataracts detected and will determine the end-point of the recruitment period.  

**Timing of assessments:**

 Most babies are discharged by 24 hours: we have taken the time period for assessment of within 72 hours of birth because this is the NIPE standard. We agree the vast majority of babies may spend less than 24 hours in the maternity unit. We have discussed the logistics of having two different screeners undertake parallel NIPE and neocam screening ward rounds with each participating unit and all can comply. Given the pressures on staffing, we are seeking to enrol and consent the majority of parents online antenatally so that parents will have had time to consider whether to participate at a less stressful time and the workload for staff on the delivery ward is minimised. 

 Why not undertake neocam screening at 6-8 week check?: The reasons why we feel the neocam check should take place at the neonatal  physical examination are as follows:

- In our experience the 6-8 week check is often delayed until 9 weeks to coincide with vaccinations, this potentially leaves very little time to organise onward specialist referral.
- The findings from the 6-8 week infant physical examination are not uploaded to a national database or audited. This study relies on data linkage with the S4N database to collect the red-reflex evaluations, therefore the neonatal screening timepoint has to be used. 
- Babies for the NIPE check are concentrated in one place for the neonatal examination and then disperse to GP surgeries for the infant examination. It would not be feasible to train thousands of GP practice staff and provide the number of prototype cameras required. 

**The two tests:**

 Risk of contamination between evalutions:

 The protocol states that different screeners will be undertaking the standard red-reflex test and neocam screening. Specific site training will  underline the importance of the screeners not discussing the results with the parents or each other until both evaluations are uploaded. We are pragmatic about the order in which the assessments are undertaken to give pressurised staff more flexibility.

 False positives and false negatives:

 Figure 2. (study flow chart) shows the method of identifying false positives and false negatives from either evaluation. Each evaluation will be compared to the gold-standard (drawn from data linkage) to give the number of false positive and negatives for each screening test. These figures will be used for the sensitivity and specifity calculations. 

 There may be occasions when both tests are falsely negative. In these cases diagnostic and procedure codes relating to cataract will be present in the HES data retrieved 12 or months after screening. It is possible that, in these cases, the cataract developed after birth. An expert panel, drawn from the Trial Steering Committee will review the neocam images in these cases to determine if there was any indication of cataract in the neonatal period. 

**Bench Testing:**

 The neocam device has had quite extensive testing in childhood populations. Correct use is important to the capture of good quality images. Just as with red-reflex assessment, the screening evaluation requires an "on-axis" examination at the correct distance. Some modifications have been made in version 2 of the prototype to promote its correct use. It is possible and an interesting thought that a future device could take a series of images co-axially and then at eccentric angles to enable an experienced clinician to determine the location of the cataract in the lens. 

 This study does not include a health economic evaluation but it will provide the data to determine if the potential improvement in visual outcomes due to earlier detection and intervention warrant a change in the screening recommendations.

# Reviewer report: Reviewer response for version 1

**DOI:** 10.3310/nihropenres.14501.r29282

**Published:** 2023-06-12

**Authors:** Anthony T. Moore

**Affiliations:** 1 Department of Ophthalmology, University of California San Francisco, San Francisco, California, USA

This study relates to the important subject of how best to carry out screening for congenital cataract in the newborn. This is currently performed using ophthalmoscopy through a natural pupil, by paediatricians or midwives. Congenital cataract is rare, and most screeners will not have had much experience in detecting cataract with this approach and most do not feel confident in identifying the target condition. It is evident from the studies cited in this paper that false positive referrals and missed cases are common. The use of a specifically designed screening device has the potential to greatly improve detection rates. Early detection is important as delayed treatment of significant congenital cataract leads to worse visual outcomes. The authors preliminary studies of a prototype device in eye clinics in Cambridge and Africa have shown that such digital imaging is superior to red reflex assessment. The aim of this study is to test this approach in the 'real world’ situation in NHS maternity units.

 The paper is well written, and the authors have established a well thought out protocol. It is important that they plan to choose maternity units serving diverse ethnic communities as detection of cataract using the red reflex is more challenging in infants with more pigmented fundi. It is also very important that the digital screener and the individual carrying out the red reflex assessment are different and that the results are not shared before both tests have been completed and results documented. This is a challenging aspect of the protocol which the authors plan to deal with by specific staff training. 

 The power calculations and statistical analysis seem very reasonable. Overall, the protocol is well thought out and achievable. One point that may need clarification is the study end point. The trial completion date is set to be 6 months after the last image has been taken. A previous study (reference 5) assessing the use of the red reflex to detect cataract found that 33% of children with congenital cataract were detected late so that they were not seen by an ophthalmologist until after 12 months of age. These would be missed in the planned study if analysis of NHS records finished before 12 months. It is not clear from the protocol when the end point for trawling NHS records will be, but clearly will need to be at least until 12 months of age.

Is the study design appropriate for the research question?

Yes

Is the rationale for, and objectives of, the study clearly described?

Yes

Are sufficient details of the methods provided to allow replication by others?

Yes

Are the datasets clearly presented in a useable and accessible format?

Not applicable

Reviewer Expertise:

Pediatric Ophthalmology

I confirm that I have read this submission and believe that I have an appropriate level of expertise to confirm that it is of an acceptable scientific standard.

# Response

**Published:** 2023-06-15

**Authors:** Louise Allen

**Affiliations:** Cambridge University Hospitals NHS Foundation Trust, UK

The authors thank Professor Moore for his review of the DIvO study protocol and agree that improving the early detection of congenital cataract is of global importance in preventing treatable childhood blindness. Many of the participating maternity hospitals will be located in cities, chosen both because of their high birthrate and the level of ethnic diversity in the population they serve.

 A very large number of babies will be recruited to the study and, given the pressure on ophthalmic and maternity services, we are using linkage to diagnostic and procedure codes in HES data as the gold-standard. Although not currently specified in the protocol, we agree that data linkage should occur at least 12 months after screening in order to reduce the risk of missing late cataract presentations. The babies screened early in the recruitment period will have up to 24 months of HES data collection. This will maximise the accuracy in determing the absolute sensitivity and specificity of each screening test.
